# *Halioticida noduliformans* infection in eggs of lobster (*Homarus gammarus*) reveals its generalist parasitic strategy in marine invertebrates

**DOI:** 10.1016/j.jip.2018.03.002

**Published:** 2018-05

**Authors:** Corey Holt, Rachel Foster, Carly L. Daniels, Mark van der Giezen, Stephen W. Feist, Grant D. Stentiford, David Bass

**Affiliations:** a Pathology and Microbial Systematics, Centre for Environment, Fisheries and Aquaculture Science (Cefas), Barrack Road, Weymouth, Dorset DT4 8UB, United Kingdom; b Biosciences, University of Exeter, Stocker Road, Exeter EX4 4QD, United Kingdom; c The National Lobster Hatchery, South Quay, Padstow PL28 9BL, United Kingdom; d The Natural History Museum, Cromwell Road, Kensington, London SW7 5BD, United Kingdom

**Keywords:** *Halioticida noduliformans*, *Homarus gammarus*, *Haliphthoros*, Oomycete, 18S rRNA gene

## Abstract

A parasite exhibiting Oomycete-like morphology and pathogenesis was isolated from discoloured eggs of the European lobster (*Homarus gammarus)* and later found in gill tissues of adults. Group-specific Oomycete primers were designed to amplify the 18S ribosomal small subunit (SSU), which initially identified the organism as the same as the ‘*Haliphthoros*’ sp. NJM 0034 strain (AB178865.1) previously isolated from abalone (imported from South Australia to Japan). However, in accordance with other published SSU-based phylogenies, the NJM 0034 isolate did not group with other known *Haliphthoros* species in our Maximum Likelihood and Bayesian phylogenies. Instead, the strain formed an orphan lineage, diverging before the separation of the Saprolegniales and Pythiales. Based upon 28S large subunit (LSU) phylogeny, our own isolate and the previously unidentified 0034 strain are both identical to the abalone pathogen *Halioticida noduliformans.* The genus shares morphological similarities with *Haliphthoros* and *Halocrusticida* and forms a clade with these in LSU phylogenies. Here, we confirm the first recorded occurrence of *H. noduliformans* in European lobsters and associate its presence with pathology of the egg mass, likely leading to reduced fecundity.

## Highlights

• First record of *Halioticida noduliformans* in European lobster eggs and gills.
• Coinfection with *Lagenidium callinectes* detected in European lobster eggs.
• Phylogenetic analyses confirm ‘*Haliphthoros* sp. NJM 0034’ is *H. noduliformans*.
• Novel Oomycete-specific 18S rRNA gene PCR primers developed and tested.
• Oomycete-specific primers detect wide diversity in environmental samples.

## Graphical abstract

**Figure:**
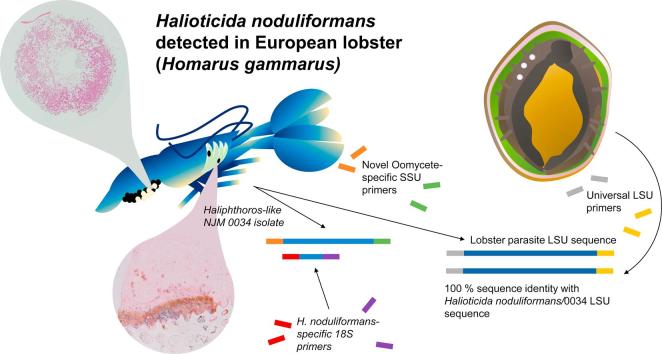

## 1 Introduction

The Oomycetes are parasitic or saprotrophic eukaryotes that group within the Stramenopile clade (Phillips et al., 2008). They include numerous taxa which infect and cause disease in aquatic invertebrates (Noga, 1990). Several Oomycete genera are known pathogens of lobsters and Crustacea in general. *Lagenidium*, has been identified as a mortality driver in larval American lobster (*Homarus americanus*) (Nilson et al., 1976) and other members of the genus have been detected in several commercially significant shrimp and crab species (Armstrong et al., 1976, Bian et al., 1979, Bland and Amerson, 1973, Lightner and Fontaine, 1973). Species belonging to the genera *Saprolegnia* and *Aphanomyces* are also notable pathogens of freshwater crayfish (Alderman et al., 1984, Diéguez-Uribeondo et al., 1994); often associated with catastrophic mortalities in natural stocks in Europe (Holdich et al., 2009).

The genus *Haliphthoros* comprises three species; *H. milfordensis, H. philippinensis and H. sabahensis.* These typically infect eggs and early life stage marine invertebrates. Infection has been described in spiny rock lobster (*Jasus edwardsii*) (Diggles, 2001), blue crab (*Portunus pelagicus*) (Nakamura and Hatai, 1995a, Nakamura and Hatai, 1995b), mud crab (*Scylla serrata, S. tranquebarica*) (Leano, 2002, Lee et al., 2017), American lobster (*Homarus americanus*) (Fisher et al., 1975), white shrimp (*Penaeus setiferus*) (Tharp and Bland, 1977), black tiger prawn larvae (*Penaeus monodon*) (Chukanhom et al., 2003), and abalone (*Haliotis* spp.) (Hatai, 1982, Sekimoto et al., 2007). Experimental challenges have also demonstrated the susceptibility of pea crab eggs (*Pinnotheres* sp.) (Ganaros, 1957, Vishniac, 1958) the European lobster (*Homarus gammarus*) (Fisher et al., 1975), ova of the blue crab (*Callinectes sapidus*) (Tharp and Bland, 1977), adult pink shrimp (*Penaeus duoraram*) (Tharp and Bland, 1977) and, the ova and larvae of brine shrimp (*Artemia salina*) (Tharp and Bland, 1977). Furthermore, *Haliphthoros* has also been isolated from the surfaces of several algae which may give an indication of its lifecycle outside of an invertebrate host infection (Fuller et al., 1964). With the exception of *H. sabahensis* in mud crab (Lee et al., 2017), all of these descriptions were solely based on the morphological characteristics of cultures isolated from the site of infection. The infection occurring in black tiger prawn (Chukanhom et al., 2003), however, was later sequenced and analysed phylogenetically (Sekimoto et al., 2007). It is therefore possible that, based on morphological descriptions alone, several of these infections could have been misdiagnosed as ‘*Haliphthoros*’ and more accurate diagnostics are required (Stentiford et al., 2014).

*Halocrusticida* (*syn. Halodaphnea*) is a closely related genus isolated from marine Crustacea, erected to contain 6 taxa belonging to the genus *Atkinsiella* (Nakamura and Hatai, 1995a). All six infect invertebrates with *A. hamanaensis, A. okinawaensis* and *A. panulirata* originally isolated from decapods (*Scylla serrata, Portunus pelagicus* and *Panulirus japonica*, respectively) (Bian and Egusa, 1980, Kitancharoen and Hatai, 1995, Nakamura and Hatai, 1995b). *Atkinsiella dubia*, a crab parasite, was the only species not to be reclassified as a member of the *Halocrusticida* (Atkins, 1954, Nakamura and Hatai, 1995a, Sparrow, 1973).

Sekimoto et al. (2007) isolated an unidentified Oomycete (NJM 0034) from white nodules in the mantle of an abalone (*Haliotis rubra*) imported to Japan from southern Australia. The pathogen most closely resembled a species of *Haliphthoros* based on characteristic morphological features such as hyphal fragmentation by cytoplasmic restriction. However, zoosporogenesis, which has traditionally served as the principle method of species identification to discern between *Haliphthoros* and its close relatives, was not observed. Upon discovery of the unidentified NJM 0034 isolate (herein referred to as 0034), Sekimoto et al. (2007) analysed three different gene loci; the ribosomal small subunit (SSU), the ribosomal large subunit (LSU) and the cytochrome *c* oxidase subunit II (cox2). In the SSU and cox2 phylogenies, 0034 branched just prior to the divergence of Peronosporales and Saprolegniales, separately from the other members of *Haliphthoros*. In the LSU phylogeny, 0034 formed a clade with ‘*Haliphthoros* sp. *NJM 0131*’, originally isolated from black tiger prawn (Chukanhom et al., 2003, Sekimoto et al., 2007). Muraosa et al. (2009) later described a second abalone parasite sharing morphological characteristics with *Haliphthoros* and erected a new genus to describe the species as *Halioticida noduliformans*. *H. noduliformans* was later isolated in wild Japanese mantis shrimp (*Oratosquilla oratoria*) from Japan and cultured abalone (*Haliotis midae*) from South Africa (Atami et al., 2009, Macey et al., 2011) and found to share 100% sequence identity to the previously sequenced 0034 in the LSU gene region (Macey et al., 2011).

As part of an ongoing programme considering novel and emerging pathogens of the European lobster (*Homarus gammarus*) in the United Kingdom, we carried out a histopathology and molecular diagnostic survey of lobsters displaying cloudy/discoloured eggs. We designed and applied new Oomycete-specific SSU PCR primers to reveal the presence of 0034 associated with the egg pathology, and generated LSU sequences from the lobster pathogen to determine whether it was the same as that in *Haliotis rubra* in Japan (Sekimoto et al., 2007). We also designed and tested 0034-specific SSU primer sets for use as molecular diagnostic tools. Our SSU analysis confirmed that 0034 cannot belong to the genus *Haliphthoros* and has no directly related SSU sequence types.

## 2 Material and methods

### 2.1 Sample collection

#### 2.1.1 Animal sampling

**Fig. 1:**
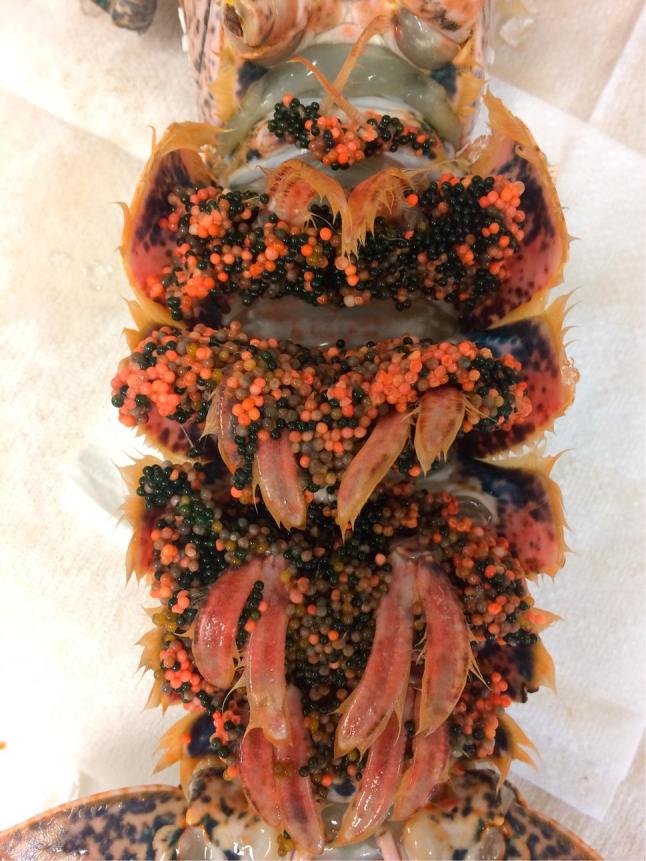
Gross pathology of infected eggs of *Homarus gammarus*. Pale, discoloured eggs observed in brood clutch of berried hen. Black eggs indicate healthy, uninfected eggs.

From July 2015 to October 2016, 323 egg bearing female lobsters were obtained from various fishermen and wholesale facilities around Cornwall and the Isles of Scilly, United Kingdom, originally recruited to take part in a larval rearing program at the National Lobster Hatchery, Padstow (UK). The landing of egg bearing females was carried out under authorisation granted by the Cornwall Inshore Fisheries and Conservation Authority (IFCA). During this period, a total of 21 animals developed abnormal egg colouration (Fig. 1) (6.5% of the total number of animals that entered the hatchery). Eighteen of the suspect 21 animals were maintained in wholesaler tanks for up to 7 days prior to transport to the hatchery. The remaining three were chilled and immediately transported. Animals that developed pathological signs of infection (n = 21) had spent between 24 and 106 days within the hatchery tank system. In order to understand the nature of the disease, animals were anaesthetised under ice for up to one hour, depending size. Heart, hepatopancreas (HP), gonad, gut, muscle, gill and eggs were removed using sterile dissecting equipment and fixed for DNA extraction, histopathology, and transmission electron microscopy. Six eggs from a subset of animals were cut in half so that histological and molecular analysis could be applied to the same individual egg. From 4th to 9th July 2016, an additional 17 egg bearing lobsters were collected on landing, from wholesalers in the south of Cornwall and processed in the same manner as above. These animals did not enter any holding tanks and are herein referred to as ‘wild’. Wild lobsters were chilled on landing and sampled that same day.

#### 2.1.2 Environmental sampling

Littoral marine, brackish water and sediment samples were collected from Newton’s Cove and the Fleet Lagoon (Weymouth, SW England) by Hartikainen et al. (2014), together with agricultural soil samples (Gosling et al., 2014), and freshwater samples from the River Avon (Bickton) and California Lake (Berkshire) (Hartikainen et al., 2016).

### 2.2 Histology

Lobster egg samples were fixed in Davidson’s Seawater Fixative for 24–48 h before transferring tissues to 70% industrial denatured alcohol (IDA). Cassettes were processed using a Leica Peloris Rapid Tissue Processor and subsequently embedded in paraffin wax. Sections were cut using a rotary microtome set at 3 µm thickness, adhered to glass slides and stained using a standard haematoxylin and eosin protocol. Slides were screened for any abnormal pathologies using a Nikon Eclipse light microscope and NIS imaging software at the Cefas Laboratory, Weymouth.

Hyphal staining was carried out following a Grocott-Gomori methanamine silver nitrate staining protocol. Slides were de-waxed and rinsed, followed by oxidation in 5% aqueous chromic acid for 1 h. Slides were then washed and rinsed in 1% aqueous sodium bisulphate for 1 min to remove excess chromic acid, washed again and subsequently placed in the incubation solution (5% sodium tetraborate, distilled water, silver solution (5% aqueous silver nitrate, 3% aqueous methenamine)), pre-heated to 50–60 °C and covered in foil, for 10 min. Stain development was checked after 5 min. This was followed by several washes in distilled water, toning in 0.1% gold chloride for 3–4 min and rinsing in distilled water. Sections were then fixed in 2% sodium thiosulphate for 2–5 min and washed under running water before counterstaining with light green dye (light green SF, acetic acid, water) for 20 s and mounting.

### 2.3 DNA extraction

One hundred mg of lobster tissue (or one egg) was transferred to an MPBio FastPrep (Lysing Matrix A) (MP Biomedicals, Santa Ana, CA) tube containing 250 µL of lysis buffer (SDS, EDTA) and homogenised. 100 µg/µL of Proteinase K was added and tubes were incubated overnight at 55 °C. 75 µL of NaCl along with 42 µL of 10% CTAB/0.7 M NaCl was added prior to further incubation at 65 °C for 10 min. DNA was isolated through phase separation with subsequent additions of chloroform and phenol:chloroform:isoamyl alcohol (25:24:1). DNA was then precipitated in 2× volume of cold 100% ethanol at −20 °C for 1 h, centrifuged to form a pellet and washed with 70% EtOH. The pellet was air dried before elution in molecular grade water.

For water samples, up to 100 L of water was serially filtered through 55 µm and 20 µm meshes. Twenty-five L of the filtered water was later serially passed through 3 µm and 0.45 µm filters. The filtrand was dried and DNA extracted using the MoBio PowerSoil DNA extraction kit (MoBio, Qiagen, Carlsbad, CA).

### 2.4 Primer design

Universal Oomycete primers were designed by manually inspecting an alignment of 215 Stramenopile sequences that spanned the 18S rRNA gene: Oom278F (5′-CTATCAGCTTTGGATGGTAGGA-3′) and Oom1024R (5′-CTCATACGGTGCTGACAAGG-3′), producing an amplicon of around 750–800 bp. The 0034-specific primers were also designed using the Stramenopile alignment with added sequence data generated from infected lobster tissue: Hali_312_F2 (5′-TGGTTCGCCCATGAGTGC-3′) and Hali_415_R1 (5′-CACAGTAAACGATGCAAGTCCATTA-3′) giving a product of ∼100 bp.

### 2.5 PCR and sequencing

PCR amplification was performed in 50 µL volumes using 10 µL of Promega 5× Green GoTaq Flexi Buffer, 5 µL of MgCl_2_, 0.5 µL of each primer (1 µM), 0.5 µL of DNTPs, 0.25 µL of GoTaq DNA Polymerase, 32.25 µL of molecular grade water and 2.5 µL of template DNA. Initial denaturation was carried out at 94 °C for 2 min. This was followed by 30 PCR cycles: denaturation at 94 °C for 1 min, annealing at 64.5 °C (Oomycete) and 67 °C (0034) for 1 min and extension at 72 °C for 1.5 min (Oomycete) and 10 s (0034), followed by a final extension at 72 °C for 5 min before being held at 4 °C.

PCR products from gill and egg tissues were directly sequenced. Amplification of the environmental samples were conducted in 20 µL final volumes with 1 µL of template DNA and was completed at the Natural History Museum, UK. The thermal cycler program was adjusted (95 °C for 5 min, 30 cycles of 95 °C for 1 min, 55 °C for 1 min and extension of 1 min 15 s at 72 °C, with a final extension at 72 °C for 10 min). Amplicons generated from environmental sampling were pooled according to habitat type (soil, marine, freshwater) and cleaned using polyethylene glycol (PEG) ethanol precipitation. Purified amplicons underwent A-tailing to improve cloning efficiency and were subsequently PEG-cleaned once more.

Clone libraries were created using the StrateClone kit (Agilent Technologies, Santa Clara, CA, USA). 32 clones from each habitat type were Sanger sequenced using the M13 forward primer at NHM. LSU gene fragments were amplified using the LSU-0021 (5′-ATTACCCGCTGAACTTAAGC-3′) and LSU-1170R (5′-GCTATCCTGAGGGAAATTTCGG-3′) following the concentrations and conditions described by Macey et al. (2011).

Sequences generated by the study are available in GenBank: accession numbers MH040872-MH040907 (Fig. 3).

### 2.6 Phylogenetic tree construction

Sequenced amplicons were added to the collection of full-length SSU Oomycete sequences with a Labyrinthulomycete outgroup. Distinct OTUs were defined as having at least one nucleotide difference in two variable regions of the gene. Those that did not satisfy this criterion were considered duplicate sequences and grouped together. Closest BLAST hits for each amplicon generated were included before aligning using the multiple sequence alignment program (MAFFT Version 7; (Katoh and Standley, 2013) and the E-INS-I iterative refinement method. The resulting alignment was used to produce a maximum likelihood phylogenetic tree inference using RAxML-HPC BlackBox version 8 (Stamatakis, 2014) on the CIPRES Science Gateway (Miller et al., 2010) using a generalised time-reversible (GTR) model with CAT approximation (all parameters estimated from the data). A Bayesian consensus tree was constructed using MrBayes v 3.2.5 (Ronquist et al., 2012). Two separate MC^3^ runs with randomly generated starting trees were carried out for 5 million generations, each with one cold and three heated chains. The evolutionary model used by this study included a GTR substitution matrix, a four-category auto-correlated gamma correction, and the covarion model. All parameters were estimated from the data. Trees were sampled every 1000 generations. The first 1.25 million generations were discarded as burn-in (trees sampled before the likelihood plots reached stationarity) and a consensus tree was constructed from the remaining sample.

### 2.7 *In-situ* hybridisation (ISH)

One hundred µl of hybridisation probes were generated using 20 µL of Promega 5× Green GoTaq Flexi Buffer, 10 µL of MgCl_2_, 2 µL of each primer (Oom278F and Oom1024R), 10 µL of DIG-labelled dNTPs, 1 µL of GoTaq DNA Polymerase, 49 µL of molecular grade water and 6 µL of template DNA. Amplification was performed using the previously mentioned thermal cycler settings.

Slides mounted with suspect wax sections were de-waxed as above and air-dried. De-waxed slides were then treated with 100 µg/ml Proteinase K in H_2_O for 15 min at 37 °C in an opaque box soaked in 5× saline-sodium citrate (SSC) buffer (trisodium citrate, NaCl, water). The slides were then incubated in 100% IDA for 5 min and subsequently rinsed in 2× washing buffer (20× SSC, Urea, BSA). Gene Frames (Thermo Fisher Scientific) were mounted on to each slide and 300 µL of probe in a 1 in 2 dilution with hybridisation buffer (100% formamide, 50% dextran sulphate, 20× SSC, 10 mg/mL yeast tRNA, 50× Denhardt’s solution) was added. Slides were then denatured at 95 °C for 5 min and hybridised overnight at 40 °C. Gene Frames were removed and slides were washed with 2× washing buffer, preheated to 40 °C, for 15 min. Hybridisation was blocked with a one hour incubation using 6% skimmed milk powder in Tris buffer. Slides were then incubated with an Anti-Digoxigenin antibody diluted in Tris buffer (1/300 dilution) for one hour at room temperature. Antibody was removed and slides were washed before staining with nitroblue tetrazolium and 5-bromo-4-chloro-3-indolyphosphate (NBT/BCIP) solution. Stained slides were then washed and counter-stained with Nuclear Fast Red before mounting and examination under light microscopy.

## 3 Results

### 3.1 Clinical signs

Infected eggs often appeared white, pink or grey relative to uninfected eggs (Fig. 1). Upon dissection, necrotic lesions were also commonly observed within the gill chambers of infected animals. Copepod parasitism within the gill chamber was observed in all animals.

### 3.2 Histopathology

**Fig. 2:**
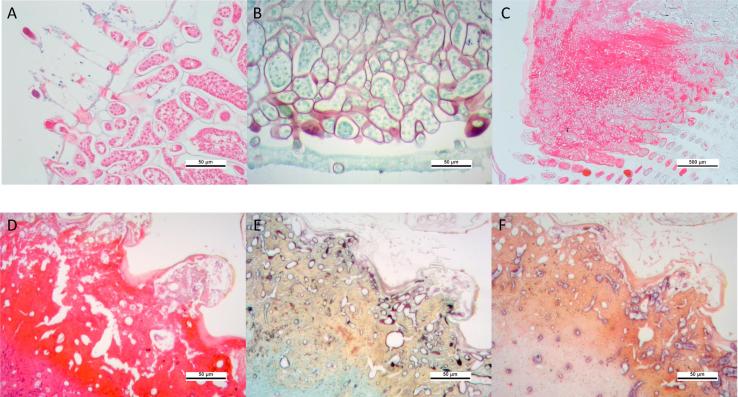
Histological sectioning of infected tissues. Light microscopy images of 3 µm tissue sections. A – Hyphae protruding from the surface of the egg. Scale bar = 50 µm. B – Silver staining of the hyphal cell walls within egg tissue. Scale bar = 50 µm. C – Low magnification image of infected gill tissue showing loss of structure and replacement with inflammatory cells and melanisation. Scale bar = 500 µm. D – Melanised lesion showing multinucleate nature of the hyphae ramifying through gill tissue. Scale bar = 500 µm. E – Silver staining of hyphal cell walls within the melanised lesion of the gill. Scale bar = 50 µm. F – *In-situ* hybridisation labelling of *H. noduliformans* using universal-oomycete SSU probes. Scale bar = 50 µm.

Egg samples from 8 out of the original 21 animals showed abnormal pathology (38% of animals in total) (Fig. 2A and B). Eggs showed a reduction or complete lack of egg yolk protein and were instead filled with large, hyphal structures. In some cases, thalli made up the entire egg mass and structures were seen protruding out of the egg membrane, potentially representing zoospore discharge tubes (Fig. 2A). Hyphae were irregular in shape and multinucleated.

Gill samples from 5 out of the 21 (24%) animals showed similar thallic structures (Fig. 2C–F). Nine of the 21 gills showed evidence of an immune response characterised by the presence of haemocyte aggregation (not shown) and melanisation (Fig. 2C and D). Hyphal cell walls were stained with silver (Fig. 2E). *In situ* hybridisation with general *Oomycete* SSU probes demonstrated the localisation of the gene target in infected tissues (Fig. 2F).

### 3.3 Molecular characterisation of the 18S ribosomal SSU in infected eggs

**Fig. 3:**
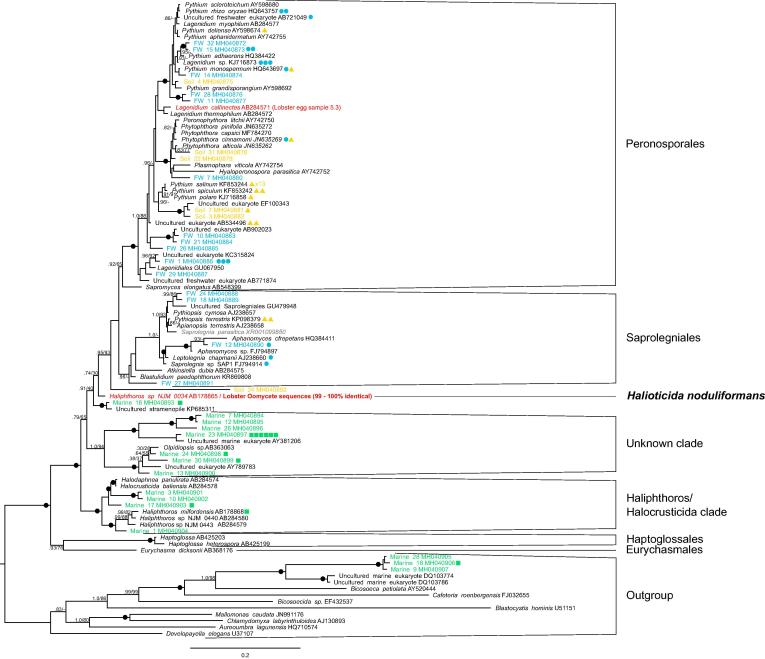
SSU gene phylogeny of the Oomycete class. Bayesian phylogeny indicating the range of oomycete diversity detected using Oomycete-specific SSU primers. Shapes accompanying tip labels indicate number of environmental samples grouped with each OTU. Circle = freshwater sample (blue), triangle = soil sample (yellow) and square = marine water sample (green). Red tip labels indicate sequences derived from lobster tissue. Grey highlights cultured, positive control. Nodes labelled with black circles indicate Bayesian/Maximum likelihood (%) support of over 0.95/95. With the exception to nodes surrounding the *Haliphthoros/Halocrusticida* clade, only support greater than 0.8/75 is annotated. (For interpretation of the references to colour in this figure legend, the reader is referred to the web version of this article.)

Oomycete-specific PCRs on all but three of the 13 eggs from the initial group produced positive PCR products (∼800 bp). Sequences obtained from excised positive bands were 99–100% identical to the *Haliphthoros* sp. NJM 0034 GenBank entry (AB178865.1). Both positive control DNA samples, *Aphanomyces invadans* and *Saprolegnia parasitica*, also amplified. Further individual egg samples (30 eggs from 5 individuals) were each bisected; one half used for histological analysis, the other for molecular analysis. All the eggs containing hyphal structures were PCR-positive using Oomycete-specific primers. In some cases, histology-negative samples produced a positive but weaker PCR product. All amplicons were sequenced and all but one of the histology positive samples produced a sequence identical to the 0034 sequence. The remaining egg, (sample 5.3) along with two histology negative samples produced PCR products which, when sequenced, showed 98–99% sequence identity with *Lagenidium callinectes* (AB284571) (Fig. 3).

Several of the histology and Oomycete PCR-positive samples were tested using the 0034-specific primer set and produced a positive amplicon of around 100 bp. Additionally, the *Lagenidium*-positive egg, sample 5.3, produced a positive, but very weak PCR product with the 0034-specific primers. Sequence data from the 0034-specific primer set confirmed the additional presence of this lineage.

### 3.4 Environmental sequencing using oomycete-specific primers

To test the specificity of the Oomycete primers we used them to amplify DNA extracted from a range of environmental samples: 16 samples from filtered coastal sea and brackish water, 24 samples from agricultural soil, and 48 samples from filtered freshwater. Eighty of the 88 environmental samples (90.9%) amplified using the Oomycete primers: 16/16 of the marine water samples, 22/24 of the soil samples and 42/48 of the freshwater samples. These sequences clustered into 71 operational taxonomic units (OTUs), which branched across the full range of Oomycete diversity as shown in Fig. 3. Thirty seven of these were identical or very similar to GenBank sequences using the same grouping criterion as described in the methods. The other 34 OTUs were novel and are indicated by FW, Soil, and Marine prefixes in Fig. 3.

Twenty six of the OTUs generated in this study grouped within the Peronosporales (12 FW, 10 Soil and two in both FW and Soil), 7 in the Saprolegniales (6 FW, one Soil) and one Soil sample (Soil 24) branched prior to the divergence of these two orders (Fig. 3). All the sequences generated from marine water samples also branched before the radiation of the Peronosporales and Saprolegniales. Three out of the 31 OTUs generated from marine sampling branched outside of the Oomycete radiation, near the Bicosoeca.

**Fig. 4:**
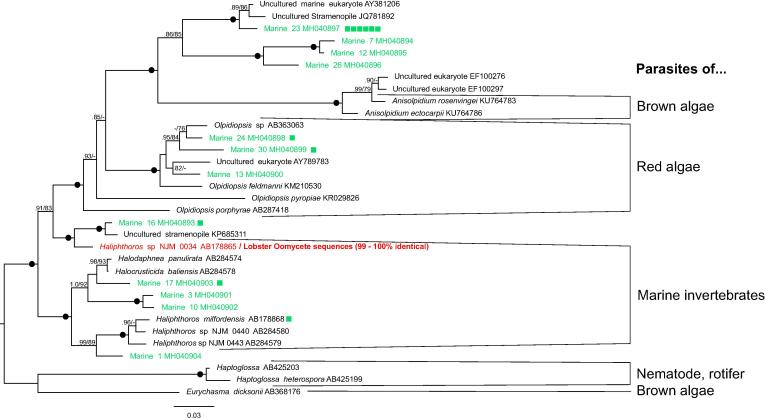
SSU gene phylogeny of the lineages surrounding NJM 0034. Bayesian phylogeny of NJM 0034 and its close relatives. Accompanying (green) squares indicate number of environmental samples grouped with that OTU. Red tip labels indicate sequences derived from lobster tissue. Nodes labelled with hollow circle indicate Bayesian/Maximum likelihood (%) support of over 0.95/95. Nodes showing support greater than 0.8/75 are annotated. (For interpretation of the references to colour in this figure legend, the reader is referred to the web version of this article.)

In phylogenetic analyses of a comprehensive taxon sampling of early-branching oomycete diversity, including 0034, *Haliphthoros, Halocrusticida, Olpidiopsis* and *Anisolpidium*, lineages cluster to some extent according to known host (Fig. 4). Four OTUs branch in a clade with the brown algae parasites *Anisolpidium* spp., three OTUs form a clade with the red algae parasites *Olpidiopsis*, and a further four OTUs in a clade with *Haliphthoros, Halocrusticida,* and *Haliphthoros* (parasites of marine invertebrates), including one grouping strongly with (AB178868) *Haliphthoros milfordensis*.

### 3.5 Phylogenetic relationships of the *Haliphthoros*-like samples

Twenty two out of the 27 sequences generated from lobster egg samples were 99–100% similar to *Haliphthoros* sp. NJM 0034 (AB178865). In ML and Bayesian phylogenetic analyses of the consensus sequence (Fig. 3), this lineage branched separately from the three other described *Haliphthoros* sequences (AB178868, AB284580, AB284579) and as a sister to the Saprolegniales but without strong support (Bayesian PP 0.74; ML bootstrap 40%). The remaining three sequences grouped with AB284571 (*Lagenidium callinectes*) (98–99% identity) isolated from marine crustacea (unpublished). Two further low-quality sequences were not analysed. LSU PCR amplification of three heavily infected eggs produced an amplicon of around 1 kb in length. Sequences generated from the isolation and purification of these products aligned with *Halioticida noduliformans* sequences (GU289906, AB506706, AB285230, AB285227) and 0034 (AB178866) with 99–100% identity. Phylogenetic analysis of the LSU region by Macey et al. (2011) showed how this *H. noduliformans* sequence branches alongside *Haliphthoros* and *Halocrusticida* species.

### 3.6 Follow-up health screen of wild lobsters

Histology of 17 wild lobster tissues did not show any abnormalities or Oomycete-related pathology. No amplicons were generated when Oomycete and *H. noduliformans* (*0034*) primers were applied to the eggs and guts. However, 5 out of the 17 gill samples weakly amplified using the Oomycete primers. Three of these tested positive for *H. noduliformans* using the species-specific primer set. There was no evidence of infection by histology in any of these samples other than the copepod parasitism of the gills as observed in the previous group of animals.

## 4 Discussion

### 4.1 Phylogenetic position of *Halioticida noduliformans*

In this study, we confirm the presence of the Oomycete pathogen *Halioticida noduliformans* as an egg parasite of the European lobster (*Homarus gammarus*). By application of improved Oomycete diagnostic primers and, by phylogenetic analysis of the amplicon derived from these primers applied to infected lobster eggs, we show that the parasite is also the same as the abalone pathogen 0034 (previously described as a *Haliphthoros* sp. NJM 0034) (Sekimoto et al., 2007). Isolation and amplification of the SSU region of the parasite from a number of eggs produced amplicons that shared 99–100% identity with 0034 (AB178865). This sequence has only previously been associated with diseased abalone in Japan (Sekimoto et al., 2007). Despite similar morphological characteristics, 0034 did not group with *Haliphthoros milfordensis* (*AB178868*) from black tiger prawn (*Penaeus monodon*) or *Haliphthoros* sp. (*AB284580, AB284579*) isolated from marine Crustacea in our SSU phylogenetic analyses and in already published analyses using the same marker gene (Beakes and Sekimoto, 2009, Sekimoto et al., 2007). The lobster egg parasite instead branched before the radiation of the more derived Saprolegniales clade. We therefore agree with the suggestion by Sekimoto et al. (2007) in that, although 0034 shares morphological similarities to *H. milfordensis* (both in terms of their wet mount observations and our histological sectioning), the isolate is clearly distinct from already described *Haliphthoros* species.

LSU rRNA gene phylogenies provide further insight into the position of the 0034 sequence type. The LSU Maximum-Likelihood phylogeny of Sekimoto et al. (2007) showed the original 0034 isolate branching as a sister to the *Haliphthoros milfordensis NJM 0131* strain (AB178869). In Macey et al.’s (2011) LSU phylogeny, 0034 is apparently identical to *Halioticida noduliformans* (GU289906); an Oomycete also isolated from nodules in the mantle of abalone and described as a causative agent of abalone tubercle mycosis disease, which causes significant mortalities in South Africa (Macey et al., 2011). There is no available corresponding SSU sequence belonging to *H. noduliformans* to allow the comparison of both gene markers however, it is likely that 0034 is *Halioticida noduliformans*, based on its LSU sequence and phylogeny and that the *Halioticida, Haliphthoros* and *Halocrusticida* genera are mutually related, together comprising parasites of aquatic invertebrates. LSU amplicons from our own isolate were identical to *Halioticida noduliformans* sequences isolated from both shrimp and abalone along with the 0034 isolate.

### 4.2 Pathology of *Halioticida noduliformans* and its relatives

To our knowledge, this is the first report of *Halioticida nofuliformans* in the European lobster or any host species from the United Kingdom and Europe. Very few references exist in terms of the histopathological descriptions of *H. noduliformans* and its closely related Oomycetes, such as *Haliphthoros.* Atami et al. (2009) offered the first histological descriptions of *H. noduliformans* in their shrimp host. They described the presence of hyphae in the gill filaments and base of those filaments. In our lobster hosts, we first detected the pathogen in discoloured egg samples. Infiltration of the egg had resulted in a mass of vegetative hyphae and the breakdown of the egg yolk protein within. Infection in adult tissues was similarly confined to the gills where growth was likely halted by the surrounding areas of melanisation; a key defence mechanism of the host. It should be noted however, that gill fouling may have contributed to the presence of necrotic tissue. No other negative health effects were observed, however, severe melanisation and subsequent necrotic lesions may well interfere with ecdysis or compromise respiratory function (Diggles, 2001, Fisher, 1977, Fisher et al., 1975). Macey et al. (2011), who reported the pathogen in abalone, also conducted a histological examination. They describe large numbers of hyphae penetrating the affected areas. However, in contrast to our own observations, where infected lobsters showed vast areas of melanisation, they note that there was ‘very little inflammation and in most cases no reaction zone’. *Haliphthoros* pathology in juvenile spiny rock lobster (*Jasus edwardsii*) shows a similar histology of the gills with the presence of multinucleate hyphae within the filaments. Hyphae and melanised lesions were also observed within the leg musculature and hepatopancreas (Diggles, 2001).

Low levels of *Halioticida noduliformans* were detected by PCR in the gills from our wild lobster health screen. However, we did not observe any pathological evidence of an infection. It is likely that adult lobsters in the wild are better able to combat the pathogen and infected eggs are prematurely dispersed to make way for the next brood (Leano, 2002). Although we do not understand its effect on the wild population, *H. noduliformans* and other similar pathogenic Oomycetes are likely to pose an increased threat to hatchery and/or aquaculture based lobsters and other invertebrates in culture situations. Intensive culture systems/sub optimal culture situations can cause physiological stresses which can increase the disease susceptibility of cultured organisms (Robohm et al., 2005). With increasing food demands and the continual growth in the world’s aquaculture industry, it is estimated that by 2030, 62% of consumed seafood will come from a farming environment (World Bank, 2013). It is therefore becoming increasingly important to better understand the health risks associated with such a shift and identify simple means in which we can detect and monitor them within these environments. That is particularly true of the Oomycetes and, more specifically the *Halioticida/Haliphthoros/Halocrusticida* clade, as they have demonstrated their ability to dramatically affect commercially important invertebrate species.

### 4.3 Oomycete-specific PCR primers

The cytochrome *c* oxidase subunits (cox) and internal transcribed spacers (ITS) have been suggested as DNA barcodes for the Oomycetes. However, these loci can be problematic for phylogenetic reconstruction, which is an important element of the interpretation of amplicon diversity (metabarcoding) data (Hartikainen et al., 2014). Choi et al. (2015) report that the ITS regions can contain large insertions exceeding 2500 bp for some species which will introduce biases in PCR amplification. Furthermore, together with an insufficient reference database, cox1 amplification does not identify all known Oomycete lineages (Choi et al., 2015). The authors demonstrate how amplification of the sequence region spanning the cox2 gene and the hypervariable cox2-1 spacer amplified all the lineages tested (n = 31) and therefore suggest that the cox2 region is better suited as a gene marker. However, these primers were not tested through means of an environmental survey and were only applied to individual lineages belonging to the Peronosporales.

LSU primers have also been used to analyse the molecular characteristics of the Oomycetes and, based on its ability to separate *Halioticida* sequences within the Haliphthoraceae, the D1/D2 region of the LSU has been suggested as a useful marker to discern between members of this family (Muraosa et al., 2009). Although the cox2 and LSU gene regions have proven beneficial in the identification of the Haliphthoraceae and the Oomycetes in general, their reference databases are not as extensive as that of the SSU gene marker. The SSU primers that we present here will facilitate better phylogenetic comparisons to be made as comparative gene sequences are more readily available. Environmental testing of the primer set has indicated their ability to detect a wide phylogenetic range of Oomycetes across all sample types tested (freshwater, marine water and soil). Thus we were able to detect *H. milfordensis* for the first time in a UK marine water sample. We have also developed a second primer set that is specific to *Halioticida noduliformans*. Using a combination of these primers, we detected cases of co-infection with *Lagenidium* in several lobster egg samples. It is possible that *H. noduliformans* infection commonly occurs in tandem with other pathogenic Oomycetes as previously reported in mangrove crab; where co-infection with *Lagenidium callinectes, Haliphthoros milfordensis* and *Halocrusticida baliensis* caused mortality rates of nearly 100% in tanked larvae (Hatai et al., 2000).

## 5 Conclusions

We provide the first evidence of infection of European lobsters (*Homarus gammarus*) by *Halioticida noduliformans* causing a destructive pathology of the eggs. To our knowledge, this is also the first report of the parasite in any animal collected from European waters. Potentially due to the unavailability of *Halioticida* and *Halocrusticida* SSU sequences, the AB178865 sequence does not resolve the phylogenetic positioning of this parasite in SSU trees. However, LSU analysis confirms its clustering within the Haliphthoraceae clade, which also contains the *Haliphthoros* and *Halocrusticida* genera.

Incidence of *Halioticida noduliformans* in the European lobsters not only demonstrates its ability to impact animals outside of its known hosts (abalone and Japanese mantis shrimp) but also, highlights the far-reaching geographical distribution of the pathogen, which has not been previously reported in Europe. This relatively newly discovered Oomycete has proven its ability to impact commercially important species and may pose a threat to future aquaculture efforts. Based on its similarity and relatedness to the genus *Haliphthoros*, it is possible that *Halioticida noduliformans* can impact a range of invertebrate species (as does *Haliphthoros milfordensis*) and therefore further work is required to highlight the extent of its host range and subsequent effects on the hatchery and aquaculture industry.

The general Oomycete and *H. noduliformans-*specific primer sets we have developed during this study should better facilitate the identification of this and other potentially problematic Oomycetes, and allow the exploration of other susceptible host species. They have been subject to environmental testing on a range of different sample types and have demonstrated their ability to identify a diverse spectrum of species that span the entire Oomycete diversity.
